# Nutritional interventions in cancer management: a comprehensive scoping review

**DOI:** 10.3389/fonc.2026.1806580

**Published:** 2026-07-10

**Authors:** Grace Taiwo Otitoju, Uju Maryanne Onuorah, Chinemerem Henry Ugo, Olawale Otitoju, Jennifer Molokwu

**Affiliations:** 1 Department of Molecular and Translational Medicine, Paul Foster School of Medicine, Texas Tech University Health Sciences Center, El Paso, TX, United States; 2 Department of Pharmacy and Health and Nutrition Sciences, University of Calabria, Rende, Italy; 3 Department of Nutrition Sciences, University of Alabama, Birmingham, AL, United States; 4 Department of Biochemistry, Federal University Wukari, Wukari, Nigeria; 5 Department of Family and Community Medicine, Paul Foster School of Medicine, Texas Tech University Health Sciences Center, El Paso, TX, United States

**Keywords:** cachexia, oncology nutrition, oral nutritional supplements, prehabilitation, quality of life

## Abstract

**Background:**

Malnutrition and treatment-related catabolism undermine outcomes in oncology. This study aimed to characterize nutritional modalities, outcomes, and contextual effectiveness in cancer management.

**Methods:**

Following a predefined scope, a comprehensive search was conducted across three academic databases to identify relevant literature. Eligible study designs for inclusion were randomized controlled trials, pilot/feasibility studies, and matched cohort studies in adults with cancer that reported patient/clinical outcomes. Data were charted on population, intervention components, comparators, outcomes, feasibility, and further synthesized narratively.

**Results:**

Fifteen studies conducted in countries such as Canada, Japan, Mexico, Spain, and India were included. The interventions were clustered as follows: (i) dietitian-led counselling with quantified energy/protein targets; (ii) oral nutrition supplements such as whey protein, ω-3/EPA-enriched formulas, and adapted foods; (iii) dietary patterns (modified Atkins); and (iv) multimodal prehabilitation including nutrition plus exercise/psychosocial, and sometimes anti-inflammatory medication. During chemotherapy, eicosapentaenoic acid (EPA)-enriched oral nutritional supplementation (ONS) improved appetite, intake, and treatment tolerability, whereas survival remained unchanged. Cachexia programs were feasible and safe, but adherence to supplements lagged behind exercise and medication. Culturally appropriate foods were also found to enhance acceptability and quality of life.

**Conclusions:**

Nutritional therapy is a feasible and clinically meaningful component of cancer care. Impact varied by context and fidelity; however, harmonized outcomes, adherence optimization, and cost-effectiveness data are needed to inform commissioning, scale-up, and evaluation strategies.

## 1 Introduction

Nutrition plays a central role in the cancer care continuum, influencing prevention, treatment response, treatment tolerance, survivorship, and palliative care. The World Cancer Research Fund/American Institute for Cancer Research identifies diet and physical activity as major modifiable drivers of cancer risk and outcomes, emphasizing the need for stronger causal inference and better integration of mechanistic and implementation research (1–3). Malnutrition, sarcopenia, and cachexia are common during treatment and are consistently linked to poorer survival and quality of life (QoL).

Recent Spanish outpatient evidence further reinforces the need for routine nutrition assessment in oncology care. In locally advanced or metastatic solid cancer patients, malnutrition risk varied by treatment context and tumor site, with higher risk among patients receiving combined chemotherapy, radiotherapy, and immunotherapy than among those receiving immunotherapy alone (de la Torre-Montero et al., 2025). This supports earlier nutrition screening and setting-specific intervention pathways across outpatient oncology services.

A range of nutritional strategies, including dietary pattern modification, individualized medical nutrition therapy (MNT), oral supplementation, and enteral or parenteral nutrition, are used to preserve physiological function, reduce treatment-related toxicity, and support adherence to treatment (4, 5). Post-diagnosis nutrition may improve survival, treatment tolerance, QoL, and symptom burden, but evidence is limited by reliance on observational studies, inconsistent measurement, and variable implementation and equity reporting (6–10). Collectively, these findings point to a systems perspective in which nutrition interacts dynamically with tumor biology, treatment modalities, and health service delivery from prehabilitation through active treatment to survivorship and end-of-life care (1, 6, 11). Despite this expanding evidence base, existing reviews remain highly segmented and organized by cancer site, dietary pattern, symptom domain, or care phase. Prevention-oriented syntheses rarely connect with treatment-era evidence. Additionally, survivorship or palliative reviews emphasize feasibility and symptom relief without employing a unified taxonomy or a consistent outcome framework (7, 9, 12, 13). Quantitative summaries addressing specific outcomes, such as fatigue and QoL, demonstrate benefits but lack generalizability across cancers and interventions (10, 14).

To address these gaps, this scoping review aimed to map the breadth of nutritional interventions examined across the cancer care continuum, characterize the outcomes assessed, and identify how effects and feasibility vary by cancer type, treatment modality, and care setting.

## 2 Methods

This scoping review was conducted in accordance with the methodological guidance outlined in the Cochrane Handbook for Systematic Reviews of Interventions and the PRISMA Extension for Scoping Reviews (PRISMA-ScR) (15, 16). This review adopted the Population-Concept-Context (PCC) framework to ensure comprehensive coverage of the literature on nutritional interventions applied in cancer management across the care continuum. The primary objective of this design was to map the breadth, nature, and characteristics of existing research rather than estimate effect sizes or assess the risk of bias. Accordingly, no critical appraisal of the quality of individual studies was undertaken.

The review followed a structured, multi-step approach involving explicit eligibility criteria, a multi-database search strategy, and standardized data charting procedures. Screening and selection were performed independently by two reviewers, and discrepancies were resolved through consensus. A PRISMA-ScR flow diagram was used to show how studies were identified, screened, assessed for eligibility, and finally included. Although the review was not formally registered, we implemented several methodological safeguards, including dual screening, clear inclusion criteria, and systematic data extraction, to support transparency and reproducibility.

### 2.1 Study eligibility criteria

The PCC framework was used to identify empirical studies of nutrition-focused interventions in human cancer populations across all care settings, while excluding non-empirical or non-nutritional studies. Peer-reviewed studies evaluating dietary patterns, medical nutrition therapy, oral supplementation, enteral or parenteral nutrition, or fasting/fasting-mimicking approaches in individuals with cancer were eligible for inclusion in the setting and care phases. **Table 1** details the inclusion and exclusion criteria applied in this study.

**Table 1:** Eligibility criteria according to the PCC framework.

Item	Inclusion criteria	Exclusion criteria
Population	Adults with any cancer type/stage; all sexes	Animal/*in vitro* studies; pre-malignant conditions only
Concept (Intervention)	Nutrition-focused interventions: dietary patterns; dietitian-led medical nutrition therapy; oral supplements (e.g., protein, omega-3, micronutrients, probiotics); enteral/parenteral nutrition; fasting/fasting-mimicking regimens	No nutrition component; exercise-only or pharmacologic-only interventions; solely mechanistic nutrient biomarker studies without an intervention
Context	Any healthcare or community setting; any country income level; any care phase (prehabilitation, active treatment, survivorship, palliative)	Not applicable
Study Designs	RCTs, quasi-experimental studies, cohorts, case–control, cross-sectional, mixed-methods, implementation/effectiveness studies	Narrative reviews, editorials, letters, protocols without results, conference abstracts without full text
Outcomes	Any of: nutritional status/body composition; treatment tolerance/toxicity; symptoms; quality of life; functional status; survival/progression; biomarker/metabolic endpoints; feasibility/adherence	Outcomes unrelated to patient or clinical endpoints (e.g., laboratory-only without clinical linkage)
Publication Type	Peer-reviewed journal articles	Preprints, theses, reports, guidelines, books/chapters, grey literature
Language	English	Non-English
Timeframe	2010-2024	Studies published outside this timeframe

### 2.2 Information sources

The retrieval process was restricted to peer-reviewed literature indexed in three databases selected for their comprehensive coverage of oncology, clinical nutrition, and allied health: MEDLINE/PubMed, Embase (Ovid), and CINAHL (EBSCOhost). No gray literature sources were consulted. The search was limited to English-language, peer-reviewed human studies published between 2010 and 2024. All records were exported to Zotero, a reference manager, for deduplication. Deduplication was followed by independent dual screening at the title/abstract and full-text stages, with consensus resolution.

### 2.3 Search strategy

A series of database-specific strategies was developed, combining controlled vocabulary (MeSH/Emtree/CINAHL Headings) and free-text terms for the Population-Concept-Context (PCC) elements (cancer, nutrition interventions), with a focus on sensitivity over specificity. The application of filters restricted the results to human subjects, English-language publications, and peer-reviewed studies. Strategies were piloted and refined to ensure the retrieval of relevant studies across intervention classes. **Table 2** lists the search strings used for each database.

**Table 2:** Search string.

Database	Search string
MEDLINE/PubMed	((“Neoplasms”[Mesh] OR cancer*[tiab] OR neoplasm*[tiab] OR tumor*[tiab] OR tumour*[tiab]) AND (“Nutrition Therapy”[Mesh] OR “Diet”[Mesh] OR “Diet Therapy”[Mesh] OR “Enteral Nutrition”[Mesh] OR “Parenteral Nutrition”[Mesh] OR “Fasting”[Mesh] OR nutrition*[tiab] OR “medical nutrition”[tiab] OR diet*[tiab] OR supplement*[tiab] OR enteral[tiab] OR parenteral[tiab] OR fasting[tiab] OR “fasting-mimicking”[tiab])) AND (english[lang]) AND (humans[mesh])
Embase (Ovid)	(‘neoplasm’/exp OR cancer*:ti,ab,kw OR neoplasm*:ti,ab,kw OR tumor*:ti,ab,kw OR tumour*:ti,ab,kw) AND (‘nutrition therapy’/exp OR ‘diet’/exp OR ‘diet therapy’/exp OR ‘enteral nutrition’/exp OR ‘parenteral nutrition’/exp OR ‘fasting’/exp OR nutrition*:ti,ab,kw OR ‘medical nutrition’:ti,ab,kw OR diet*:ti,ab,kw OR supplement*:ti,ab,kw OR enteral:ti,ab,kw OR parenteral:ti,ab,kw OR fasting:ti,ab,kw OR ‘fasting-mimicking’:ti,ab,kw) AND [english]/lim AND [humans]/lim AND [article]/lim
CINAHL (EBSCOhost)	(MH “Neoplasms+”) OR (TI cancer* OR AB cancer* OR TI neoplasm* OR AB neoplasm* OR TI tumor* OR AB tumor* OR TI tumour* OR AB tumour*) AND ((MH “Nutrition Therapy+”) OR (MH “Diet+”) OR (MH “Diet Therapy+”) OR (MH “Enteral Nutrition+”) OR (MH “Parenteral Nutrition+”) OR TI nutrition* OR AB nutrition* OR TI “medical nutrition” OR AB “medical nutrition” OR TI diet* OR AB diet* OR TI supplement* OR AB supplement* OR TI enteral OR AB enteral OR TI parenteral OR AB parenteral OR TI fasting OR AB fasting OR TI “fasting-mimicking” OR AB “fasting-mimicking”)) Limiters: English Language; Peer Reviewed; Human

### 2.4 Study selection

**Figure 1:**
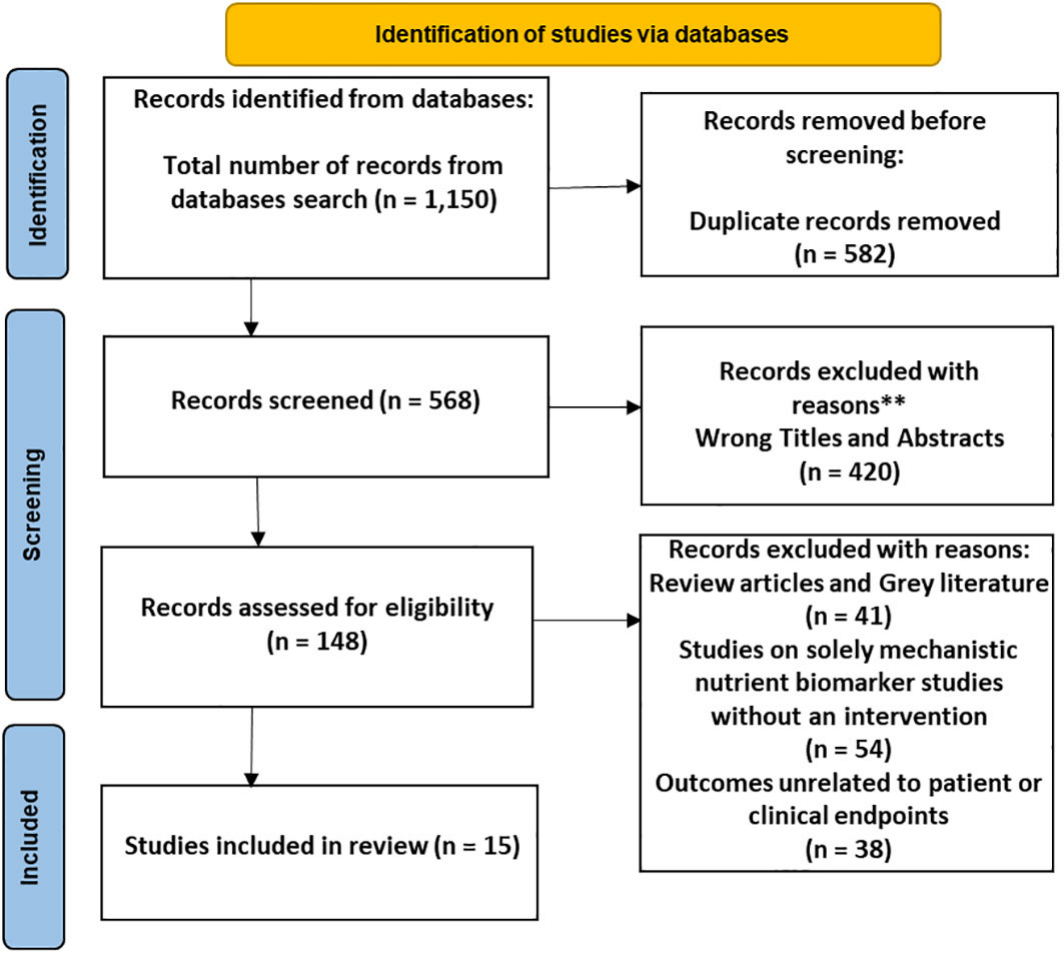
PRISMA-ScR detailing the study selection process.

The search yielded 1,150 records screened. After removing 582 duplicates, 568 titles and abstracts were screened by two reviewers. Of these, 420 were excluded based on title/abstract. A total of 148 full texts were assessed, of which 133 were excluded based on criteria including review/grey literature (n=41), biomarker-only mechanistic studies without an intervention (n=54), and studies lacking patient/clinical outcomes (n=38). Fifteen studies met the inclusion criteria. The decisions and their rationales were meticulously documented to populate the PRISMA flowchart (**Figure 1**).

### 2.5 Data extraction and synthesis

Data were extracted from each eligible study using a standardized charting form, piloted on a sample of studies to ensure consistency and clarity. The extracted variables included bibliographic details, study characteristics (design, country, sample size), population and cancer type, intervention components (nutrition modality, dose, duration, dietitian involvement), co-interventions, comparators, and reported outcomes. Additional fields captured feasibility, adherence, implementation context, and equity-related markers, when available. The data were extracted by Grace Taiwo Otitoju (GTO) and independently checked for accuracy by Uju Maryanne Onuorah (UMO). Discrepancies were reconciled through joint review and consensus discussions among the reviewers. This process ensured reliability and minimized data abstraction errors in the study.

The synthesis was descriptive and mapping-oriented rather than effect estimating. A numerical summary was produced, encompassing counts by year, region, cancer type, intervention class, and outcome domains. In addition, a narrative thematic grouping by intervention class was prepared, categorizing dietary patterns, medical nutrition therapy, oral supplementation, enteral/parenteral nutrition, fasting/fasting mimicking, and linking to outcome domains.

## 3 Results

### 3.1 Study characteristics

Fifteen studies encompassing randomized trials, pilots, feasibility designs, and matched cohorts, with participants drawn from Canada, the USA, the UK/Norway (Europe), Spain (Europe), Japan, Mexico, and India, were included in this review (**Table 3**). The study population comprised surgical candidates with colorectal, bladder, hepato-pancreato-biliary, and non-small cell lung cancer (NSCLC) and advanced cachexia. The interventions encompassed dietitian-led counselling with energy/protein targets, whey or EPA supplements, adapted ice-cream, and the Atkins diet, frequently within multimodal prehabilitation (comprising exercise/relaxation and celecoxib for cachexia). The outcomes encompassed various parameters, including functional capacity, presence of complications, duration of hospitalization, tolerance to treatment, body weight and composition, appetite, and quality of life. Adherence levels varied, but the treatment’s safety was deemed acceptable.

**Table 3:** Included studies.

Study (year)	Population & setting	Design & N	Nutrition intervention (type, components, dose, timing, duration; dietitian-led)?	Co-interventions	Comparator	Outcomes measured	Key findings (direction, signals)
(17)	Adults with advanced cancer in an interdisciplinary oncology rehabilitation service (Canada); outpatient palliative-leaning context.	Prospective cohort; N = 188 (single-center).	Individualized nutrition-rehabilitation: dietitian assessment, tailored counselling to meet energy/protein needs; oral nutritional supplements (ONS) if intake inadequate; delivered during program; ~10–12 weeks; dietitian-led with ≥q2-week visits.	Structured exercise therapy; symptom and psychosocial support embedded in team care.	Usual/standard care outside the program (implicit).	Functional performance, symptom burden, QoL, weight/other nutrition indices.	Participation was associated with improved function/QoL and stabilization of nutrition status among completers; ~51% maintained or gained weight; pragmatic signals rather than causal estimates.
(18)	Colorectal cancer (CRC) patients awaiting resection (Canada); preoperative setting.	Double-blind RCT (pilot); N = 48.	Whey-protein ONS as part of prehabilitation; supplement contributed roughly ~20% of prescribed protein; ~4 weeks pre-op + 4 weeks post-op; supervised by a dietitian with intake logging.	Aerobic/resistance training; anxiety-reduction/relaxation.	Placebo ONS within the same prehab framework.	6-minute walk distance (6MWD), postoperative recovery metrics (complications/LOS), nutrition markers.	Greater pre-op functional gains vs control; favourable recovery trends (small–moderate effects); nutrition component feasible and acceptable.
(19)	CRC surgical candidates (Canada); tertiary surgical center.	Interventional pilot; N = 87 (prehab 42; control 45).	Trimodal prehabilitation nutrition: dietitian-led counselling with 30–35 kcal/kg/day target; whey isolate to reach ~1.2 g/kg/day protein; started several weeks pre-op; median ~33 days to surgery.	Supervised exercise; anxiety-management training.	Standard postoperative rehabilitation without prehab.	Time to functional recovery, 6MWD, complications, LOS.	Earlier functional recovery and better pre-op capacity vs rehab-only; suggests added value of pre-op nutrition alongside exercise.
(20)	CRC on a potentially curative pathway (UK); mixed inpatient/outpatient phases.	Non-randomized feasibility; 22/84 (26%) recruited; 15 (18%) completed.	Lifestyle/weight-management diet programmed: structured behavior-change counselling, diet quality improvement, portion and protein guidance; dietitian involvement; 3 face-to-face + 9 phone sessions, median ≈5.5 h contact across pre-op, recovery, post-treatment phases.	Physical activity coaching; behavioral support.	Standard care without a structured lifestyle programmed.	Feasibility/uptake, diet/PA behaviors, weight, QoL.	Feasible and acceptable; trend to ↑ activity; no consistent changes in weight/QoL; perioperative timing and comorbidity burden constrained delivery.
(21)	Patients undergoing radical cystectomy (Canada); perioperative programmed.	RCT; 70 randomized (35 prehab; 35 control).	Tailored nutrition counselling with protein goals; ONS where required; dietitian-led; begins weeks pre-op and stops at surgery (with peri-op reinforcement).	Exercise training; anxiety-reduction.	Standard care/post-op rehab without structured prehab.	6MWD pre-op and 4 & 8 weeks post-op; complications; LOS; readmissions.	4 weeks post-op: 6MWD decline smaller with prehab (−15.4 m) vs control (−97.9 m), p=0.014; no intervention-related AEs.
(22)	HPB (hepato-pancreato-biliary) cancer surgery candidates (Japan).	Propensity-matched cohort; prehab n=76 vs control n=76.	Pre-op nutrition therapy: dietitian assessment, counselling ± ONS; calorie/protein targeting to maintain albumin/weight; several weeks pre-op.	Supervised exercise sessions.	Historical/parallel usual care without structured prehab.	Cardiorespiratory fitness, serum albumin, complications, LOS, weight/nutrition indices.	Prehab stabilized/improved albumin, ↑ 6MWD and muscle: fat ratio; LOS shorter (median 23 vs 30 days, p=0.045); overall complication rates similar.
(23)	Lung or pancreatic cancer with cachexia (Nordic multi-center).	Phase II RCT (feasibility); 46 randomized (25 multimodal; 21 control); 41 completed.	Cachexia-focused nutrition + ONS (EPA/DHA-enriched) within MENAC concept; dietitian-led; several weeks. Component adherence: celecoxib 76%, exercise 60%, ONS 48%.	Progressive resistance/aerobic home exercise; celecoxib.	Standard care.	Feasibility (recruitment, attrition, adherence), safety; exploratory weight/lean mass, PA, QoL, appetite.	Feasible and safe; weight change favored intervention (e.g., mean +1.29% vs −3.19%); survival similar (study underpowered).
(24)	CRC patients scheduled for resection (Canada).	RCT; N≈80 analyzed.	Supervised nutrition within multimodal prehab: dietitian-led counselling, daily whey-protein ONS, protein goals; pre-op over several weeks.	Supervised exercise; anxiety-reduction strategies.	Standard care/rehabilitation without prehab.	6MWD, complications, LOS, nutrition parameters.	Improved pre-op capacity and favourable recovery signals; underscores operational feasibility of dietitian-integrated prehab.
(25)	Adult women with cancer cachexia receiving palliative care (India).	RCT; n=63 (30 intervention; 33 control).	Improved Atta (IAtta) flour 100 g/day for 6 months (mean intake ≈45 ± 11 g/day ≈180 kcal, 11 g protein) + counselling; control received counselling only; dietitian-led.	Usual palliative symptom management.	Standard palliative care without structured nutrition package.	Intake, PG-SGA, weight, MUAC, EORTC QLQ-C30 domains.	Control lost weight and MUAC; intervention stabilized weight (trend), ↑ body fat; fatigue and appetite loss improved; no AEs to IAtta reported.
(26)	Advanced NSCLC on systemic therapy (Mexico).	RCT; n=92 (46 per group).	EPA-containing ONS 2 cans/day (~237 mL each) for ~8 weeks; mean intake 1.4 ± 0.6 cans/day; dietitian oversight for adherence.	None beyond usual oncology care.	Isocaloric diet advice/ONS without EPA (per trial specifics).	Weight/lean mass, energy/protein intake; symptoms (fatigue, neuropathy); QoL.	EPA-ONS arm showed better nutritional status and higher intake with reductions in fatigue/neuropathy vs control; survival not different (per abstract).
(27)	Mixed cancers on chemotherapy without overt malnutrition (Spain).	Randomized, placebo-controlled pilot; n reported ~66 (EPA-ONS vs isoenergetic control).	EPA-enriched ONS: 2 × 240 mL/day (~295 kcal, ~16 g protein, ~1 g EPA per pack; target 2 g EPA/day); mean compliance ≈1.6 g EPA/day; dietitian-guided.	Usual care.	Isoenergetic, EPA-free ONS/control.	Chemotherapy tolerability (dose intensity, delays), weight, intake.	EPA-ONS associated with fewer dose-limiting toxicities and more cycles completed; trends to better weight maintenance; adherence acceptable.
(28)	Mixed cancer cohort, outpatient (Spain).	Interventional cohort; N≈70 (adapted ice-cream n≈39; standard ONS n≈31).	Adapted ice-cream supplement to increase energy/protein intake; palatable format to overcome anorexia/dysgeusia; dietitian involvement.	None.	Usual diet (non-supplemented).	QoL (e.g., EORTC QLQ-C30 domains), weight/BMI.	QoL improvements in selected domains and weight stabilization; highlights acceptability/palatability as adherence levers.
(29)	Advanced malignancies in a VA health system (USA).	Feasibility trial; N = 17.	Modified Atkins/ketogenic diet: ~20–40 g carbohydrate/day, adequate protein; intensive counselling; home monitoring for up to ~16 weeks; dietitian-led.	None (dietary intervention only).	Usual diet (implicit comparator).	Feasibility, safety, weight/metabolic markers, QoL.	Feasible for a motivated subset with metabolic shifts; adherence variable; safety acceptable with monitoring.
(30)	Advanced GI, NSCLC, or mesothelioma with weight loss on chemotherapy (UK; multi-center).	Four-arm RCT; N = 358 (no intervention; diet advice; ONS; advice+ONS).	Energy/protein ONS in ONS arms; standardized dietetic advice in advice arms; delivered across chemo cycles; dietitian-led.	–	No intervention; diet advice alone; ONS alone; advice+ONS.	1-year survival, weight, QoL.	No between-group differences in survival, weight, or QoL; patients surviving >26 weeks gained weight regardless of allocation.
(31)	Advanced cancer with anorexia (USA).	RCT; ~141 enrolled (~118 evaluable).	Behavioral nutrition advice: prescribed pre-meal white wine ≤15%, twice daily for ~3–4 weeks; short-term regimen with safety screening; clinician-supervised.	None.	Standard advice without wine.	Appetite scales, caloric intake, weight, QoL.	Modest orexigenic signal; weight effects limited; illustrates behavioral/dietary counselling feasibility in appetite management.

### 3.2 Settings and institutional delivery models

The included evidence should be interpreted by care setting, and not as a single uniform intervention field. Perioperative studies were mainly delivered in prehabilitation and tertiary surgical programs, where Dietitians were embedded within multidisciplinary teams and outcomes focused on recovery, functional capacity, complications, and hospital stay. Outpatient chemotherapy studies were more supplementation-focused, commonly evaluating EPA-enriched ONS, treatment tolerance, appetite, intake, and weight maintenance. Palliative and cachexia-focused studies were more practical, combining nutrition counselling, rehabilitation, symptom management, appetite support, or culturally acceptable food-based fortification. Institutional differences were evident in referral pathways, dietitian availability, access to commercial ONS, intensity of monitoring, home-based follow-up, and the availability of exercise, psychosocial, or anti-inflammatory co-interventions. Therefore, intervention effects should be interpreted as setting-dependent signals shaped by institutional capacity, treatment window, and implementation fidelity rather than as a uniform nutrition-support effect across all oncology contexts.

### 3.3 Thematic synthesis

Across the 15 studies, four main types of interventions emerged. Most programs focused on dietitian-led counseling, using specific nutritional targets, goal-setting, and follow-up via clinic visits or phone calls. Oral nutrition supplements (ONS) were widely used. Whey-based supplements appeared frequently in prehabilitation programs, while n-3-enriched formulas (EPA/DHA) were common during chemotherapy or for managing cachexia. Some interventions also incorporated adapted foods, such as high-energy ice creams, to address anorexia or taste changes.

Dietary pattern interventions were less common but included tightly supervised approaches such as modified Atkins or ketogenic diets. Multimodal prehabilitation combined nutrition therapy with structured aerobic and resistance training, and in some cases, relaxation strategies or anti-inflammatory medications used for cachexia.

Delivery models varied widely, ranging from supervised, center-based sessions to primarily home-based programs supported by logs and weekly check-ins. Interventions were typically timed to coincide with the preoperative period (2–8 weeks) or delivered during chemotherapy or palliative care, with duration aligned to the clinical treatment window.

Support strategies to promote adherence, such as dose adjustments, flavor or texture modifications, and reminder systems, were inconsistently described. In resource-limited settings, culturally familiar and pragmatic options, such as fortified flour blends in India, helped improve engagement when commercial ONS were not readily available.

### 3.4 Outcome domains and measurement landscape

The outcome assessment encompassed seven domains: The primary endpoint of perioperative trials was predominantly focused on functional capacity, specifically the 6-minute walk distance. The postoperative outcomes included complications, length of stay, and readmissions. Treatment tolerance (dose intensity, delays) was assessed in chemotherapy cohorts receiving EPA-ONS. Nutritional status or body composition was determined using weight, mid-upper arm circumference, lean mass estimates, and serum albumin levels. Cachexia studies incorporated appetite and intake diaries. Symptoms and patient-reported outcomes, such as fatigue, appetite loss, and global health, were measured using validated tools, including the European Organization for Research and Treatment of Cancer Quality of Life Questionnaire-Core 30 (EORTC QLQ-C30). Enhancements in quality of life were evident in instances of high adherence to the prescribed regimen and in the delivery of palatable treatments (e.g., adapted ice cream). Feasibility and adherence featured prominently in feasibility and phase II studies. The heterogeneity of measurement techniques persisted, with only a few studies reporting the use of standardized body composition techniques or a core outcome set. This limitation resulted in significant complications in the cross-study synthesis process. Nonetheless, the recurrence of patterns (6 MWD, intake/weight, and complication metrics) enables the mapping of data across intervention classes and care phases.

### 3.5 Contextual effectiveness and feasibility across the care continuum

Across surgical pathways, nutrition-integrated prehabilitation consistently improved preoperative function, with some studies showing signals for shorter hospital stays in matched cohorts. During chemotherapy, EPA-enriched ONS improved intake, helped maintain weight, and reduced dose-limiting toxicities, although survival outcomes remained unchanged.

In patients with advanced disease and cachexia, multimodal interventions were both feasible and safe. Still, adherence differed across components; in the multimodal cachexia trial, adherence was highest for the anti-inflammatory medication component, intermediate for exercise, and lowest for ONS, which may have influenced weight and lean-mass outcomes. Modified Atkins diets were feasible for highly motivated participants, producing expected metabolic changes but inconsistent effects on weight.

Feasibility varied by setting. Although prehabilitation periods were short, structured coaching and home-based programs supported strong engagement in high-income centers. In resource-limited settings, culturally familiar food-based strategies, such as fortified flour, helped stabilize weight and improve fatigue and appetite, with high acceptability.

Across contexts, three factors consistently shaped adherence: access to dietitians, the palatability of prescribed foods or supplements, and the intensity of monitoring. Importantly, safety profiles were uniformly acceptable, supporting the potential scalability of nutrition-focused interventions tailored to tumor type, treatment phase, and local resources.

## 4 Discussion

This scoping review demonstrates that nutrition support interventions can be most clinically appropriate during the treatment phase, depending on patient risk and institutional capacity. Dietitians’ counselling with quantified energy/protein targets was frequently incorporated into multimodal prehabilitation in perioperative oncology, and it was linked with better functional capacity and recovery signals. EPA enriched ONS tended to have beneficial effects on appetite, intake, weight maintenance and treatment tolerance in outpatient chemotherapy. Multimodal nutrition-rehabilitation and culturally appropriate food based fortification were feasible and acceptable in palliative and cachexia settings, and effectiveness of the clinical intervention was largely dependent on disease burden, adherence, level of monitoring, and local resources.

The overall survival signal is not consistent and should not be read as a lack of clinical value of nutrition support. The majority of trials included were small, short and did not have power to detect differences in survival. Tumor biology, disease stage, response to systemic therapy, tumor progression, and comorbidities significantly affect survival as well and can obscure the ability to measure the impact of nutrition support. Conversely, shorter-term outcomes, such as hospital stay, treatment tolerance, appetite, intake, and functional capacity are more proximal measures, and therefore more likely to respond over shorter timeframes during prehabilitation, chemotherapy support or cachexia care time frame. Improvements in hospital stay may likely reflect better functional reserve, earlier mobilization, improved perioperative coordination, fewer nutrition-related delays, and greater readiness for discharge, although these pathways require direct testing in adequately powered trials.

The role of pre-operative immunonutrition needs to be more clearly defined, but not overemphasized. Traditional immunonutrient formulations of arginine, omega-3 fatty acids and nucleotides were not the focus of the preoperative studies included in this review; rather these studies were predominantly concerned with the impact of dietitian-led counselling, protein targets and whey/protein supplementation. Future surgical oncology trials should compare standard protein-focused supplementation with immunonutrition-enriched formulations, stratify participants by baseline malnutrition risk, and report complications, infections, length of stay, functional recovery, adherence, and cost-effectiveness using harmonized outcome measures.

### 4.1 Implications for policy and practice

Nutritional care should be formally established as a standard, funded component of oncology care. This component would involve universal screening at diagnosis and at each treatment transition, guaranteed, reimbursed access to registered dietitians, and formulary pathways for oral nutrition supplements, including EPA-enriched options, with equity safeguards. Nutritional considerations should be incorporated into ERAS and prehabilitation protocols, with the establishment of quality indicators such as time-to-dietitian, PG-SGA completion, and adherence capture being paramount. In addition, the requirement for core outcome sets for audits should be stipulated. In settings where resources are limited, it is essential to prioritize culturally acceptable, food-based fortification measures, along with simplified protocols and telehealth for follow-up.

The implementation of a screen-triage-treat-monitor model is essential, with targets set at 30–35 kcal/kg/day and 1.2 g/kg/day protein. The deployment of dietitian-led counselling is also crucial, with the consideration of EPA-ONS during chemotherapy and multimodal strategies for cachexia. To ensure optimal patient compliance, it is recommended that adherence support, such as palatability, reminders, and remote logs, be used. Conversely, it is advisable to refrain from providing non-standard or experiential advice. It is imperative to tailor treatment to the condition’s stage, presenting symptoms, and treatment window. The outcomes of the track are to be monitored using 6MWD, PG-SGA, and EORTC QLQ-C30, with the resulting data being fed into iterative improvement cycles. Training teams in brief nutrition interventions and culturally adapted recipes is recommended to strengthen uptake and equity.

Because nutrition-support approaches differ across institutions and care settings, implementation should be adapted rather than standardized as a single model. Tertiary surgical centers may prioritize nutrition-integrated prehabilitation and immunonutrition trials; outpatient chemotherapy units should prioritize screening, dietitian referral, EPA-ONS pathways, symptom-responsive counselling, and adherence monitoring; palliative and resource-limited settings should prioritize feasible food-based fortification, appetite support, caregiver education, and simplified follow-up. This setting-specific model would make nutrition support more scalable, equitable, and responsive to the realities of oncology care delivery.

### 4.2 Strengths and limitations

The study’s strengths include its clearly articulated scope, which focuses on patient-relevant outcomes; its comprehensive multi-database search (2010–2024); its duplicate screening with reviewers and its transparent PRISMA reporting. The review integrates perioperative and advanced/cachexia contexts, maps intervention typologies, and charts feasibility, adherence, and safety, producing practice-oriented insights across diverse settings (high-income countries (HICs)/low- and middle-income countries (LMICs). Data were extracted using a standardized template to enable cross-study comparisons.

The limitations center on evidence heterogeneity, including small sample sizes, pilot/feasibility designs, inconsistent dosing/timing/fidelity reporting, and non-standardized outcomes. This heterogeneity precludes meta-analysis and reduces the precision of effects. The absence of protocol registration and formal critical appraisal reduces reproducibility and certainty. The exclusion of gray literature and pediatric studies, probable publication bias towards positive findings, and geographic bias towards high-resource centers all constrain generalizability. Few studies reported long-term or cost-effectiveness outcomes, and adherence data (especially for supplements) were inconsistently captured, complicating implementation in routine services and equitable access.

## 5 Conclusion

Nutrition therapy represents a clinically meaningful and feasible component of modern cancer care. Across fifteen studies, dietitian-led counselling with quantified energy/protein targets, often paired with whey or EPA-enriched supplements and embedded in multimodal prehabilitation, improved intake, weight stability, and preoperative functional capacity, with acceptable safety. Analysis of chemotherapy cohorts revealed that patients demonstrated enhanced tolerance to treatment and improved symptom management when administered EPA-ONS, although survival outcomes remained unaltered. Multimodal cachexia programs were found to be deliverable, albeit hindered by variable adherence. The enhancement of acceptability was particularly effective when culturally appropriate foods were used, especially in circumstances where commercial products were not readily available. Evidence gaps include a lack of standardized outcomes, intervention fidelity data, long-term effects data, and cost-effectiveness data. It is recommended that policymakers consider commissioning routine screening and guaranteed dietetic access. In addition, it is advised that healthcare professionals operationalize pathways that include screening, triage, treatment, and monitoring. These pathways should be tailored to the tumor stage and treatment window. In future trials, it is necessary to adopt core outcome sets, stratify by cancer context, and test scalable delivery models, including telehealth and food-based fortification, to optimize both reach and equity. Nutrition should be considered a fundamental aspect of treatment, rather than an afterthought in all settings.
